# Somatic mutations in solid tumors: a spectrum at the service of diagnostic armamentarium or an indecipherable puzzle? The morphological eyes looking for *BRAF* and somatic molecular detections on cyto-histological samples

**DOI:** 10.18632/oncotarget.12564

**Published:** 2016-10-11

**Authors:** Esther Diana Rossi, Maurizio Martini, Tommaso Bizzarro, Fernando Schmitt, Adhemar Longatto-Filho, Luigi Maria Larocca

**Affiliations:** ^1^ Division of Anatomic Pathology and Histology, Università Cattolica del Sacro Cuore, “Agostino Gemelli” School of Medicine, Rome, Italy; ^2^ Instituto de Patologia e Imunologia Molecular da Universidade do Porto, Porto, Portugal; ^3^ Department of Medicine and Pathology, Laboratoire National de Santé, Luxembourg; ^4^ Department of Pathology, Laboratory of Medical Investigation, University of São Paulo School of Medicine, Brazil; ^5^ Life and Health Sciences Research Institute, School of Health Sciences, University of Minho, Braga, Portugal; ^6^ ICVS/3B's, PT Government Associate Laboratory, Braga/Guimarães, Portugal; ^7^ Molecular Oncology Research Center, Barretos Cancer Hospital, Pio XII Foundation, Barretos, Brazil

**Keywords:** plump eosinophilic cell, Warburg effect, glycolysis, BRAF mutation

## Abstract

This review article deals with the analysis and the detection of the morphological features associated with somatic mutations, mostly *BRAF^V600E^* mutation, on both cytological and histological samples of carcinomas. Few authors demonstrated that some architectural and specific cellular findings (i.e. polygonal eosinophilic cells defined as “plump cells” and sickle-shaped nuclei) are able to predict *BRAF* *^V600E^* mutation in both cytological and histological samples of papillary thyroid carcinoma (PTC) as well as in other carcinomas. In the current review article we evaluated the first comprehensive analysis of the morphological prediction of *BRAF^V600E^* and other somatic mutations in different malignant lesions with the description of the possible mechanisms beneath these morphologic features. The detection of predictive morphological features, mostly on FNAC, may add helpful information to the stratification of the malignant risk and personalized management of cancers. Additionally, the knowledge of the molecular mechanism of different oncogenic drivers can lead to the organ-specific triaging selection of cases and can provide significant insight for targeted therapies in different malignant lesions.

## INTRODUCTION

The recent findings of specific morphological parameters associated with *BRAF^V600E^* neoplasms offer a promising and foreseeable insight into the morphological detection of somatic mutations supported also by the high specificity and positive predictive value of these parameters [1-6]. In this regard, this “morphological” evaluation seems to represent a valid and predictive support which may balance the difficulties in the use of DNA-based molecular analysis. Conversely, it is mostly performed with molecular platforms including different types of sequencing [3-4,7-11]. Despite their invaluable advantages, little reluctance might be ascribed to the fact that they are more inquiring, and less cost-effective for their worldwide diffusion in any laboratory [7-11].

Several studies, referring to different malignant neoplasms such as melanomas, hairy-cell leukemia (HCL), colon carcinoma (CC), ovarian low-grade serous carcinoma (LGSC), Langerhans cell histiocytosis and Erdheim-Chester disease (ECD) and glial neoplasms [12-21] have demonstrated the diagnostic and prognostic involvement of BRAF*^V600E^* mutation in the molecular mechanism of cancers.

Additionaly, in papillary thyroid carcinoma (PTC), *BRAF* *^V600E^* mutation was found to be associated with some distinctive architectural and cellular morphological features (specifically plumps cells defined by polygonal eosinophilic cytoplasms and and sickle-shaped nuclei) with significant diagnostic accuracy [1-4].

Accordingly, these promising morphological insights were underscored in a recent study pointing to the detection in *BRAF^V600E^* ovarian neoplastic lesions [5]. In this context, the authors identified a population of *BRAF^V600E^* ovarian cells, characterized by the same abundant eosinophilic cytoplasm (EC) among the “ovarian atypical proliferative serous tumours” [5]. Furthermore, owing to the histological and molecular features, the authors suggested that *BRAF^V600E^* mutation is highly likely to activate neoplastic initiation followed by a mechanism to restrain tumor progression and reach low-stage senescent disease as previously observed in vitro [5].

Peculiar morphological findings have been described in other BRAF-driven mutated cancers. In fact, hairy cell leukemia (HCL), characterized by specific morphological features (namely abundant eosinophilic cytoplasm and “hairy” membranous projections), demonstrate that *BRAF^V600E^* mutation is the disease-defining genetic event inasmuch as this mutation is present in virtually all the HCL cases but rarely in other B-cell lymphomas [16-17]. Similarly, foamy and eosinophilic cytoplasms have been appreciated in several Langerhans cell histiocytosis and Erdheim-Chester disease (ECD) harbouring *BRAF^V600E^* mutation [15].

Not surprisingly, some other neoplasms are likely to demonstrate a correlation between molecular alterations and distinctive morphological features. In fact, *PDGFRA* mutations in gastrointestinal stromal tumors (GISTs) have been found to correlate with several noteworthy morphological features (i.e. epithelioid morphology of tumor cells and tumor-infiltrating mast cells) allowing histological discrimination from GISTs with c-kit mutations. [19] Additionally, *ALK* translocations have been exclusively reported in lung carcinomas belonging to the acinar and solid predominant adenocarcinomas hystotypes [20].

In this review article we emphasize that, investigating the genetic underpinnings of the detection of morphological features link to some somatic mutations on both cytological and histological samples of different malignant entities may pave the way for obtaining an accurate knowledge of the molecular mechanism of different oncogenic drivers. Hence, this knowledge may lead to the organ-specific triaging selection of cases in order to provide important insight for future tailored targeted therapies in different malignant lesions

## BRAFV600E AND THYROID

Numerous studies have found a strict association between thyroid carcinoma and activating somatic mutations in the *BRAF* oncogene demonstrating a high prevalence of these mutations mostly in the classical papillary thyroid carcinoma (PTC) and/or its more aggressive variant (including for instance tall cell variant-TCV and sclerosing variant of PTC-SVPTC) [7-8, 21-30]. According to literature, the majority of *BRAF* mutations (more than 90%) involve the exon 15 with a final valine to glutamine substitution in the Braf protein (V600E) [21-26]. Nikiforov recognized that *BRAF^V600E^,RAS, RET/PTC* and *PAX8/PPAR*γ were associated with malignancy in almost 100% of thyroid lesions demonstrating the role of somatic mutations and/or rearrangements in everyday practice [31-35]. Given that, in thyroid oncology, *BRAF* *^V600E^* mutation is strongly associated with thyroid carcinoma mainly with the classical PTC hystotype.

Besides the diagnostic role of *BRAF^V600E^* mutation as a specific hallmark of thyroid tumorigenesis, this mutation has also provided significant insights into the prognostic unfavourable outcome of mutated carcinomas [24-27]. Despite contrasting evidence, *BRAF^V600E^* has been found in cases with higher tumor aggressiveness demonstrated by more frequent local or distant metastases, multifocality and infiltration of the peri-thyroid tissue. [24-27, 36-39]. The recognition of this subset of patients with a specific genotype and aggressive phenotype induced Finkelstein [4] and Virk [3] to analyze the correlation between *BRAF^V600E^* mutation and some specific architectural and cellular morphological parameters in the histological PTCs. Specifically this discovery included the evidence of foreseeable predictive features such as the presence of more infiltrative borders of the carcinomas, numerous psammoma bodies, well-developed features of PTC and peculiar polygonal cells with abundant and eosinophilic cytoplasms (defined as “plump cells”). In fact, Virk applied the term “plump cells” in order to define these large polygonal neoplastic cells, with nuclear features of PTCs but also with their height, less than twice the width, with squamous metaplasia and mostly with eosinophilic cytoplasms [3]. They demonstrated these peculiar cells in 72% of their *BRAF^V600E^* PTCs but also in 28% of the wild type PTCs [3]. Their yields confirmed the data previously published by Finkelstein demonstrating plump in 65% of the *BRAF^V600E^* PTCs [4].

**Figure 1:**
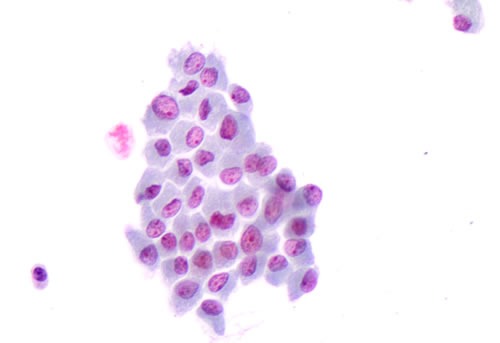
Details of the morphological features of plump cells and sickle shape nuclei in a cluster of “positive for malignancy” thyroid cells on liquid based cytology (LBC, 40X)

**Figure 2:**
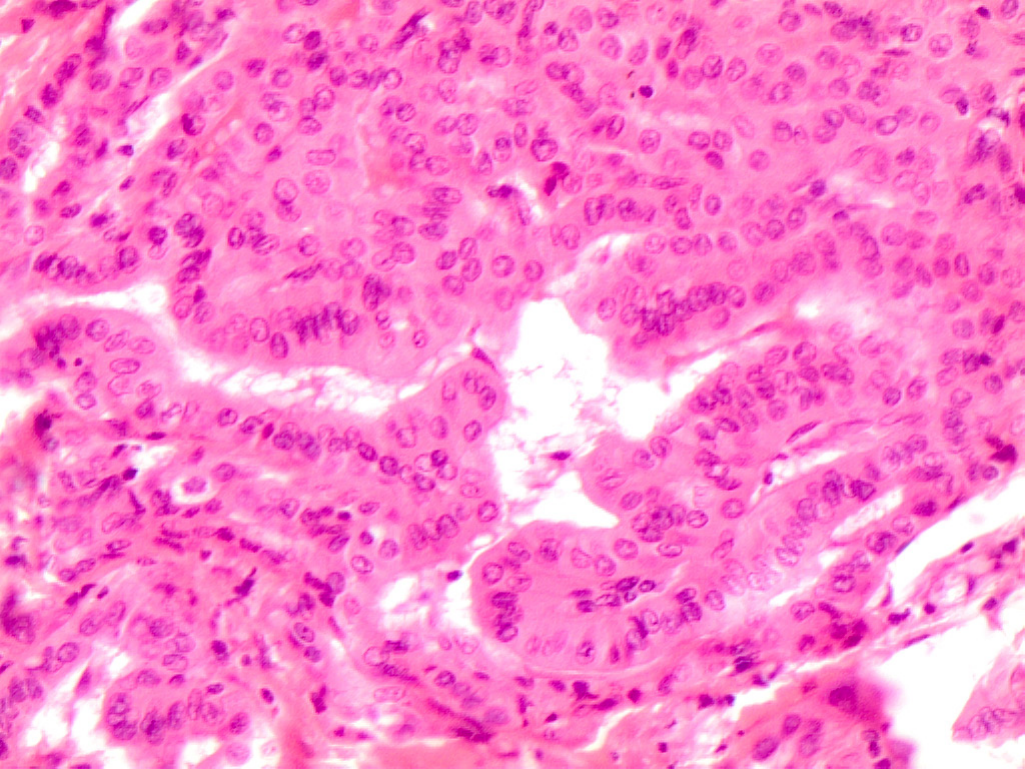
Specific histological details of polygonal cells with homogeneous eosinophilic cytoplasm (plump cells) in the bottom of the picture obtained from a Follicular variant of PTC (FVPC- H&E 60X)

Taking into account these findings, we were tempted to search for the same morphological features in our series of thyroid lesions diagnosed as positive for malignancy (favouring PTC) on cytology and processed with liquid based cytology (LBC). [1-2]. For the reason that we were searching these parameters on cytological specimens, we were likely to address our study only to the cellular features mostly directed to recognize these large polygonal eosinophilic tumor cells characterized by nuclear features of PTC and homogeneous, moderate to abundant cytoplasms. The same morphological parameters were assessed on the corresponding histological samples. In contrast with the detection of “plump cells” in 72% of Virk's *BRAF^V600E^* cases; we recognized them in 100% of the mutated cases even if with a different intensity in the eosinophilic cytoplasms which may be attributed to the use of LBC [1-2; Figure 1]. Moreover,

the quantitative definition of these cells represents an additional critical parameter. Virk and Finkelstein appraised that even focal “plump cells” are correlated with *BRAF* *^V600E^* mutation and this evidence was also found in our 12.7% of the *BRAF* *^V600E^* mutated cases with focal “plump cells” [3-4]. Additionally we had 5 out of 25 wild type cases with focal “plump cells” [1]. In our opinion, in the interpretation of focal “plump cells”, there may be a problematic semi-quantitative evaluation of the percentage score which might not be predictive of the *BRAF* *^V600E^* status. Importantly, for the first time to date, we reported the evidence of a particular and patognomonic nuclear shape in all the *BRAF* *^V600E^*cases. Specifically, these nuclei were smaller, in eccentric position and with a sickled shape which was absent in wild type *BRAF* cases [1-2; Figure 1]. The sickle-shaped nuclei, characterized by nuclear grooves and pseudo inclusions, had also irregular nuclear membranes. The same morphological parameters were recognized on the corresponding histological samples (Figure 2).

**Figure 3:**
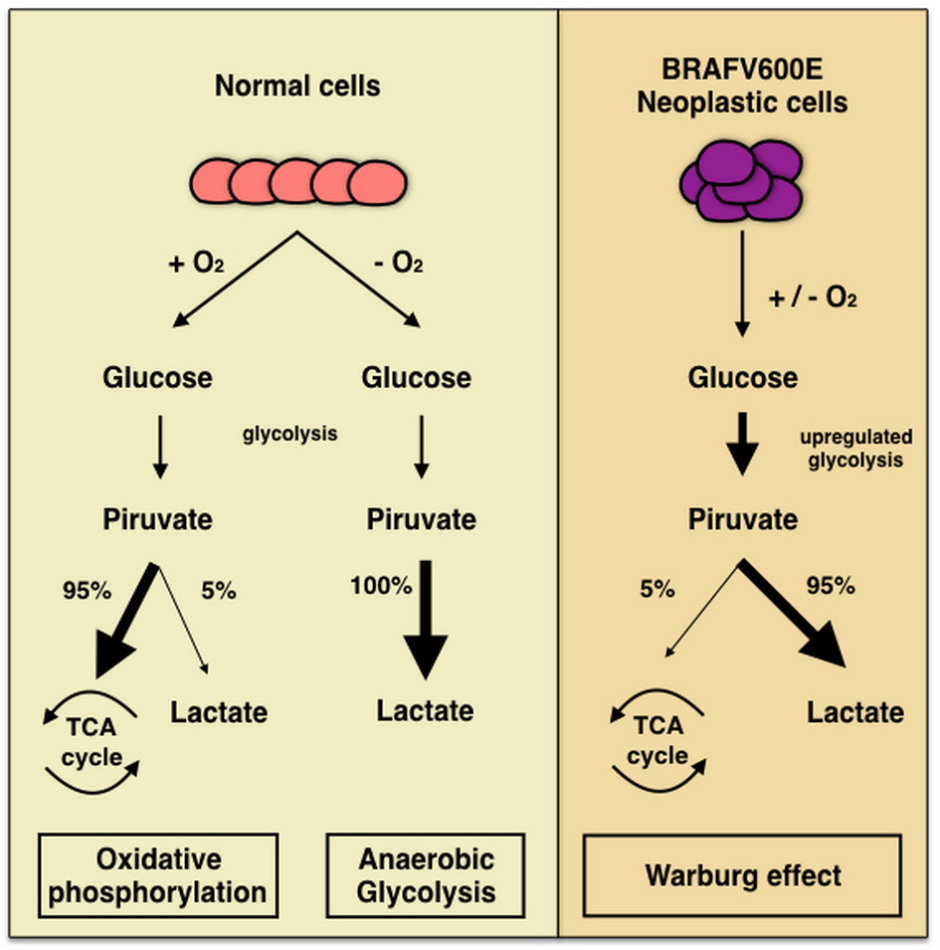
Comparative analysis of normal and neoplastic cells and “Warburg effect” pioneering the ability of cancer cells of converting glucose to lactic acid

In this perspective, the morphological evidence of altered nuclei/cytoplasm ratio (favoring a moderate/abundant cytoplasm in *BRAF^V600E^* PTCs) cannot refute the argument that mutated cancer cells had a different metabolism due to the effect of mutated oncogenes on the pathway [7,12; Figure 3].

However, all these papers, including ours, limited their analysis to acknowledge the correlation of these “plump cells” with *BRAF* *^V600E^* mutation in the PTCs without any description or study concerning the mechanisms beneath their nature [1-4].

Among authors, Hall suggested that these morphological findings are the consequence of the “Warburg effect” consistent in the ability of cancer cells of converting glucose to lactic acid [12; Figure 3]. According to their studies, Hall et al suggested that this effect may induce the activation of *RAS-BRAF-MEK-ERK* pathways which stimulate glycolysis also when there is presence of glucose [12-13, 40-41]. In this perspective, this up-regulation of glycolisis gene in *BRAF* mutated cells, may lead to an increased mitochondrial respiration justifying the abundant and eosinophilic cytoplasm of the cells [12-13, 42-43]. In this perspective, one explanation of this cytoplasm feature may be ascribed to the role of glycolysis through the accumulation of the lactate which was highlighted by the immunocytochemical analysis of the monocarboxylate (MCT) family. In fact, MCTs are involved in regulation of lactate and other monocarboxylates across cellular membranes.[44-50]. Inasmuch as glycolisis stimulates the accumulation of high amount of lactate inside cancers cells, MCTs regulate the maintenance of the intracellular PH of the neoplastic cells through the extrusion of lactate. According to literature, MCTs are transmembrane proteins including 14 members even thoughonly the first 4 MCTs,(MCT1-4), are involved in the regulation of lactate and other MCTs across cellular membranes. The role of MCTs in thyroid *BRAF^V600E^* carcinomas has not been completely defined so that their use as potential therapeutic targets needs to be demonstrated with practical application [51]. In our recent experience, we found that plump PTC cells yielded high positive predictive value (92%) and specificity (87%) for MCT1 whilst high predictive values and sensitivity for MCT4 [52].). Their expression clearly depicts that the different metabolism of *BRAF* *^V600E^* cells (involving the up-regulation of gene associated with glycolysis) may be responsible for the abundant eosinophilic cytoplasm of the plump cells [13-14]. Together, a recent study by Lee prospected the evidence of *BRAF^V600E^*mitochondrial translocation involving both a metabolic remodeling and an anti-apoptotic effect through the caspases 3-9 [13].

In fact, it has been demonstrated that this localization confers apoptotic resistance and the characteristic metabolic phenotypes associated with suppressed oxidative phosphorylation due to the bind between *BRAF^V600E^* mitochondrial interacting molecules, which have not already been, identified [34]

In this regard, a possible molecular hypothesis, which might justify the higher aggressiveness encountered in mutated PTC, seems to involve the *BRAF* *^V600E^* localization within the outer mitochondrial membrane. This evidence may imply a novel and potential therapeutic approach for the treatment of patients.

## BRAF^V600E^ MUTATION IN OVARIAN NEOPLASMS

Among the broad range of human tumors found to harbor *BRAF* mutation, ovarian neoplasms, including atypicalproliferative serous tumors (APSTs) and low grade serous carcinomas (LGSCs), account for two thirds mutated cases in different series by literature [5-6, 51-61]. However, Zeppernick examined its association with the clinicopathological characteristics in ovarian neoplasms [5-6].

**Figure 4:**
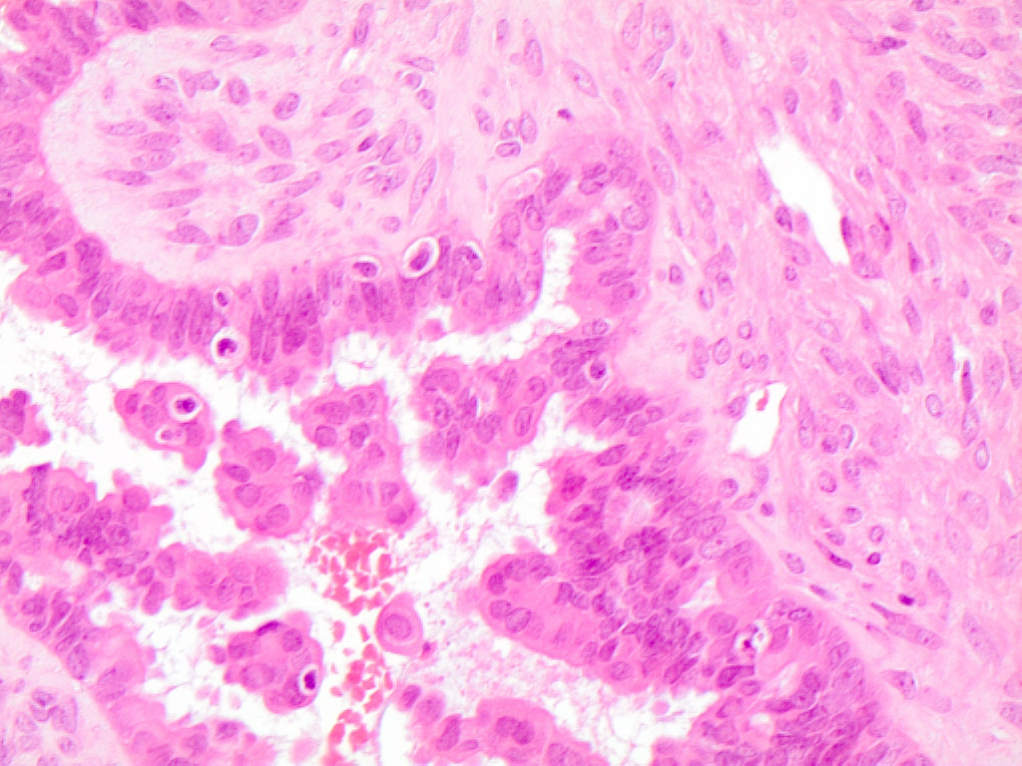
Histological details of the morphological features of plump cells on serous borderline mutated ovarian case (H&E 60X)

More specifically, Zeppernick described some specific morphological features in 71atypical proliferative serous tumors (APSTs) and 18low grade serous carcinomas (LGSCs) and therefore their association with *KRAS* and *BRAF mutations*. They demonstrated that all the *BRAF*V600E ASPTs were characterized by abundant eosinophilic cytoplasm (EC) whilst only 2 *BRAF*V600E LGSCs presented the same EC cells. Furthermore, the immunohistochemical studies revealed that these EC cells were characterized by low Ki-67 proliferation index, significant decrease in steroid hormone receptors and strong p16 expression [5]. Owing to the histological and molecular features, the authors suggested that *BRAF*V600E mutation is highly likely to activate neoplastic initiation followed by a mechanism able to restrain tumor progression and to increase low-stage senescent disease, as previously observed in vitro [6]. Despite the same morphological evidence of *BRAF*V600E mutation in both ovarian (Figure 4) and thyroid neoplasms, we hypothesize that *BRAF*V600E activating mutation may produce a different or/and opposite effect on different body sites controlled by a specific metabolism and/or hormonal status. Hence, despite the similarities of cellular features associated with *BRAF*V600E mutation, it also activates organ-specific molecular mechanisms enabling different prognostic prediction in these cancers [5-6].

Given that, the association between *BRAF*^V600E^ mutation and ovarian morphology demonstrates the pathogenesis of these specific lesions with an “oncogene-induced senescence” phenotype as described by Zeppernick, which may block the progression of the precursor (APSTs) to cancer (LGSCs). Eventually the identification of EC cells may help in planning a personalized management especially because the majority of APSTs do not progress to LGSCs.

## BRAF^V600E^ MUTATED MELANOMAS

As partially described in thyroid chapter, some of the mechanisms proposed as responsible for the morphological features in mutated PTCs had been borrowed from the literature concerning *BRAF*V600E melanomas [12, 62-73]. Haq in his papers assessed that *BRAF*V600E mutation is the most frequent genetic mutation in melanomas and the activation of the BRAF/MAPK decreases the oxidative metabolism [66-67]. As Salama and Flaherty summarized, *BRAF* V600E has garnered a great deal of attention in these years especially as the most frequent oncogenetic mechanism in up to 60% of the cutaneous melanomas [62]. Nonetheless, these authors did not underline any correlation with specific phenotype of mutated melanoma cells. However, despite this lack of evidence, some authors found a higher incidence of *BRAF*V600E in melanomas developed on body sites intermittently exposed to the sun than those arisen in areas with chronic exposure [63]. Furthermore, Kim showed an association between *BRAF*V600E mutation and different types (acral lentiginous melanoma) and spreading of melanoma (superficial or nodular melanoma)[63]

**Figure 5:**
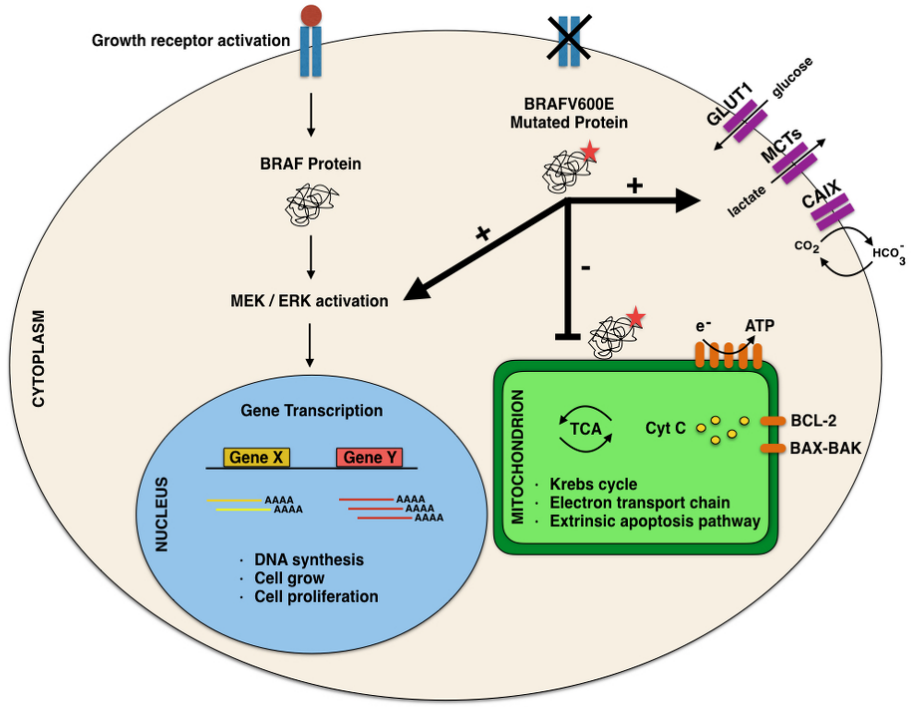
Details of the molecular mechanisms involving BRAF mutation in the different cell localizations

On the other hand, Hall assessed that most cancer cells, including melanoma cells, display a strikingly different metabolism that is generated by intrinsic and extrinsic factors [12]. These authors proved the first evidence that *BRAF*V600E mutation stimulates the upregulation of gene involved in glycolysis in mutated melanoma cell lines [12]. Therefore, their results suggested that *BRAF* activation is associated with reduction of oxidative enzymes, mitochondrial number and alterations in the lactate (increasing amount). In contrast with the data from thyroid cancer cell, Hall did not find evidence of *BRAF* correlated suppression of oxidative phosphorylation in melanoma cells. This metabolic reprogramming triggered by *BRAF*V600E is linked with the suppression of melanocyte master regulator microphthalmia-associated transcription factor (MITF) and mitochondrial master regulator (PGC1α) representing the major regulator of mitochondrial biogenesis and function [67-71; Figure 5].

Taking into account the results published in *BRAF* mutated melanomas, though unproved on thyroid PTCs, BRAF inhibition also stimulates some gene programs such as oxidative phosphorylation, mitochondrial mechanisms, and the levels of PGC1α [67-71, 74]. Given that, the activation of the BRAF/MAPK pathway may suppress levels of MITF and PGC1α and decrease oxidative metabolism [14, 72]. Data reported from melanomas cells, demonstrated that oncogenic *BRAF* promotes melanoma metastasis and aggressiveness through the down-regulation of the cyclic *GMP (cGMP*)-specific phosphodiesterase PDE5A with interaction between *RAS/RAF* and *cAMP* pathway [14,72]. Arozarena had previously demonstrated that *BRAF* V600E increases the levels of BRN2 (a transcriptor factor) in melanoma cells, which is responsible for invasiveness [64-65]. Additionally, it binds to the PDE5A promoter that is essential for the suppression of the PDE5A transcription by *BRAF* V600E. Despite the demonstration of these mechanisms in both in vivo and in vitro studies and cell lines, to date, they have not been correlated with peculiar morphological evidence on melanoma cells.

## BRAF^V600E^ IN HEMATOLOGICAL NEOPLASMS

As reported by Davies, *BRAF* oncogene was found mutated also in some specific hematological neoplasms including hairy cell leukemia but also Langerhans cell histiocytosis and Erdheim-Chester disease [75-76]. In 2014, Machnicki discussed the role of *BRAF* in hematological entities even though it has not been clearly defined [77]. Although the oncogenic role of *BRAF*V600E has been reported in lymphoid and myeloid neoplasms, it seems to be mostly restricted to some specific subtypes. Conversely, the prevalence of *BRAF* V600E mutation in chronic and acute leukemias ranges between 0 and 10% with only Gustafsson demonstrated that 20.7% of acute lymphoblastic leukemia (ALL) had *BRAF* V600E mutations [78]. Despite the fact that these *BRAF*V600E mutated cases are devoid of any morphological finding linked to the mutation, the detection itself, restricted to specific histotypes is a relevant feature as target for *ERK* pathway inhibitors. Hence, other attempts were made to determine whether *BRAF* mutations are present in multiple myeloma (MM) and plasma cell leukemia (PCL) [79-90]. Despite the low incidence of *BRAF* mutations in these entities, Andrulis found 2.8% *BRAF* V600E cases out of the 379 patients with plasma cell disorders [79].

On the other hand, the selectivity of *BRAF*V600E mutation is also clearly stated in hairy cell leukemia (HCL), an uncommon B-cell neoplasm in which *BRAF*V600E mutation represents an almost 100% disease-defining genetic abnormality [17, 86-91]. The pathognomonic features of typical hairy cells with abundant cytoplasm and circumferential distribution of fine hair-like projections on the cellular surface have been worldwide accepted [86-91]. Conversely, the fact that HCLs show the peculiar hairy cytoplasmic projections is likely to represent the morphological evidence of *BRAF*V600E mutation recalling plump cells especially for the abundant cytoplasms. On the other hand, these hairy cells are not reported in other BRAF negative B-cell leukemias [86-91]. As recently reviewed by McCarthy, the *BRAF*V600E-MEK-ERK pathway might be promoted by the production of ketones [40]. In this regard, Kang demonstrated that HMG-CoA lyase (HMGCL), involved in the ketogenesis pathway, activates the interaction between *BRAF*V600E and MEK leading to the subsequent phosphorylation of MEK1 as shown in hairy-cell leukemia and melanoma cells [41].

According to literature, this mutational status has its major correlation with the diagnosis and treatment of HCL, so as to help in the choice of targeted therapies (namely interferon-α [IF-α] and purine nucleoside analogs [PAs]) allowing a complete remission in the majority of patients. However, additional studies may be addressed to understand how ketones stimulate the interaction between *BRAF*V600E and MEK and their role in inducing the morphological findings.

Additionally *BRAF*V600E mutation was also detected in other two rare histiocytoses: Langerhans-cell histiocytosis (LCH) and Erdheim-Chester disease (ECD) characterized by a phenotype similar to CD1a^+^ S100^+^ Langerhans cells and foamy CD68^+^, CD1a^-^ S100^-^ histiocytes, respectively [15, 92-100]. Despite the controversy whether LCH and ECD are either flogistic entities or clonal neoplasms, the latter hypothesis is strongly supported by the detection of *BRAF* V600E mutation in up to half of the cases [92-98]. In the review article by Machnicki, the frequency of *BRAF* V600E ranged from 37.9% by Haroche to 68.8% by Satoh in LCH whilst it was at around 31.4% in ECD [77,100].

The high prevalence of *BRAF*V600E mutation restricted to these two histiocytoses is liable to encourage the management with vemurafenib or other ERK pathway inhibitors [100].

## BRAF^V600E^ IN CENTRAL NERVOUS SYSTEM NEOPLASMS

Several authors estimated that *BRAF*V600E represents a frequent mutation in ganglioglioma (GG) [101-105]. On the other hand, the prevalence of *BRAF*V600E mutation in other low-grade gliomas has not been clearly recognized. In their paper, MacConaill reported a single case of pilocytic astrocytoma with *BRAF*V600E mutation, whilst others pointed out that some diffuse astrocytomas (WHO grade II) harboured *BRAF* V600E mutation [101, 102, 105].

Somatic mutations and specifically *BRAF*V600E through the MAPK signaling have been reported in 60% of the pleomorphic xanthoastrocytoma (PXA) representing a rare low-grade glial neoplasm of the central nervous system mostly occurring in children and young adults [103]. Specifically largepleomorphic giant cells with some xanthomatous change and showing dense deposition of intercellular reticulin and eosinophilic granular bodies are the peculiar features that are found in these tumors. It may be hypothesized that these cytoplasmic findings can be the effect of the altered cellular mechanisms activated by the somatic mutation. In this regard, the identification of *BRAF*V600E mutations, as commonly detected in PXAs, might help in discriminating them from classical glioblastoma multiforme and/or pilocytic astrocytoma [103,104]. The suggestion that *BRAF*V600E mutations is the hallmark of both PXA and GG is also demonstrated by the evidence that ganglioglioma (GG), shares significant clinicopathologic similarities with PXA, [102-104].

Moreover, Lee, in a series of 105brain tumors (including 51dysembryoplastic neuroepithelial tumors, 14subependymal giant cell astrocytomas, 12glioblastoma with neuronal marker expression, and 28 pleomorphic xanthoastrocytomas) assessed 51% and 42.9% mutated cases in the dysembryoplastic neuroepithelial tumor and subependymal giant cell astrocytomas. This data may support and encourage the aid of specific tailored therapies [104]

## BRAF^V600E^ IN GASTRO-INTESTINAL NEOPLASMS

The evidence of morphological features associated with *BRAF*V600E mutations have been found in colon neoplasms [18, 106-110]. Specifically, recent data suggest that *BRAF*V600E mutation is one of the most frequent molecular abnormalities identified in hyperplastic polyps and sessile serrated adenomas which are early precursor lesions in the pathway of carcinogenesis [106-107]. In contrast, traditional serrated adenomas may be associated with either *BRAF*V600E or *KRAS* mutations [108]. According to Rosty, *BRAF*V600E mutation was assessed in several sessile serrated adenoma/polyps with or without dysplasia and microvesicular hyperplastic polyps with a percentage ranging from 95 to 76%. Hence, in their paper they attributed the morphological features of globet cells tubulo-villous adenoma mainly to *KRAS* mutation [109].

**Figure 6:**
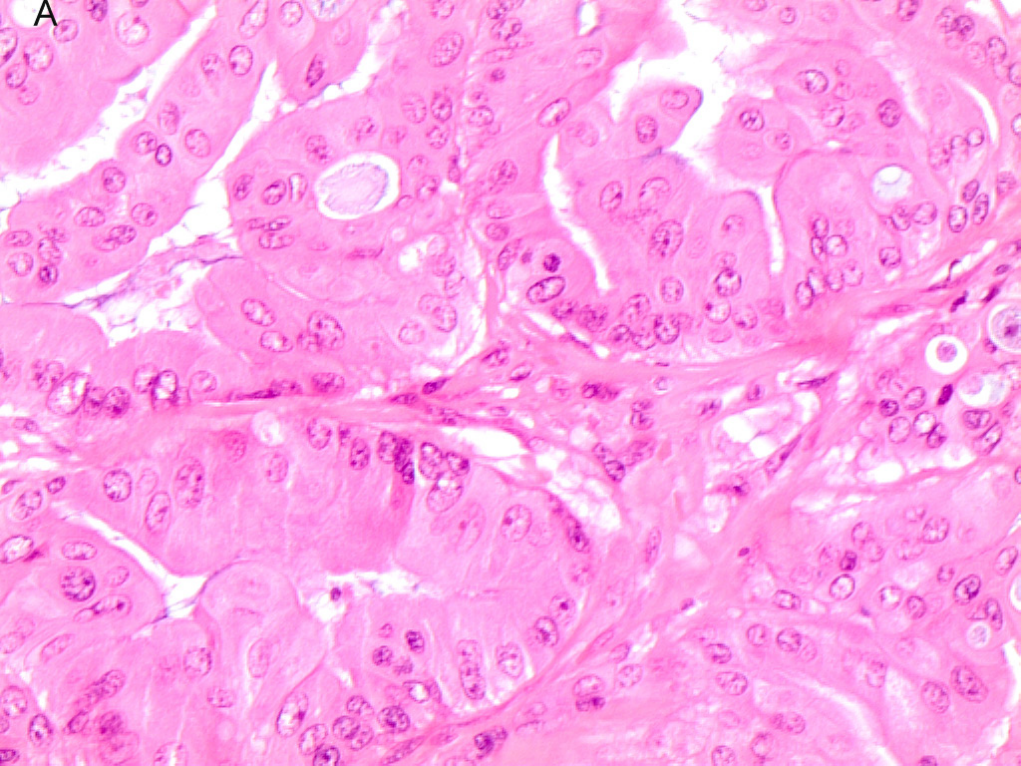
**A.** Histological details of the morphological features of cells on mutated intestinal adenocarcinoma case (H&E 60X); **B.** Histological details of the morphological features of cells on wild type intestinal adenocarcinoma case (H&E 60X).

The *BRAF* molecular correlation was also supported by the immunohistochemical positivity for VE1 suggesting a subclassification of polyps according to the mutation status. In this perspective, Mesteri proposed that the evaluation of serrated lesions with immunohistochemical or molecular *BRAF*V600E mutation may be the key to identify those lesions with higher potential to progression into *BRAF*V600E -mutated colorectal [110; Figure 6A, 6B]. As assessed in the pictures 6a-6b the presence of *BRAF*V600E mutation induced the peculiar moderate-abundant cytoplasm, which were absent in wild type adenocarcinomas. These morphological features underline their unequivocal correlation with *BRAF*V600E mutation. Additionally, as reported by Rosenbaum et al, colon carcinomas harboring *BRAF*V600E mutation are also characterized by the expression of programmed cell death 1(PD-1) and its ligand (PD-L1), microsatellite instability and a peculiar medullary morphology with frequent CD-8 positive tumor-infiltrating lymphocytes. Moreover, the PD-L1 expression in colorectal carcinomas is associated with a worse outcome as well as tumors with microsatellite instability.[111].

## NOT ONLY BRAFBRAF^V600E^ MUTATION AND MORPHOLOGICAL FINDINGS

Not only did some authors report a correlation between *BRAF*V600E mutation and morphological features but also other papers documented some specific morphological clues linked with other mutations [19, 20, 112-115]. Although in 1998, the expression of c-kit protein associated with *c-kit* mutation has been considered a diagnostic hallmark of gastrointestinal stromal tumors (GISTs), a subset of them were *c-kit* wild type [114-115]. In fact, it has been well documented that a significant proportion of gastrointestinal stromal tumors (GISTs) harbour also activating mutations of platelet-derived growth factor receptor a (*PDGFRA*). This latter mutation seems to be associated with peculiar morphological features allowing them to be discriminated from GISTs with *c-kit* mutations [19, 114-115]. Together, some authors, including Tajima, observed that GISTs with *PDGFRA* mutations are frequently characterized by epithelioid pattern, myxoid stroma, mast-cells infiltrating tumor, multi-nucleated neoplastic cells and some rhabdoid cells [19]. It stands to reason that, also among GISTs, the morphological finding may be a predictive factor of a sensitive mutation leading to personalized molecular-targeted therapy [19,114-115].

In the same perspective, mutations in the V-Ki-ras2 Kirsten rat sarcoma viral oncogne homologue (*KRAS*), epidermal growth factor receptor (*EGFR*), v-Raf murine sarcoma viral oncogene homologue B1 (*BRAF*) and translocations of the anaplastic lymphoma kinase (*ALK*) gene locus have been found in several lung adenocarcinoma (ADC) with important implications for targeted therapies [20,112]. Warth highlighted that *KRAS* mutations were more frequently found in invasive mucinous ADCs whilst *EGFR* mutations were associated with lepidic growth and micropapillary ADCs [20]. On the other hand, *ALK* traslocations were recognized as oncogene drivers only in acinar and solid ADCs whilst *BRAF* mutation in micropapillary ADCs but not in papillary and lepidic ADCs [112]. This peculiar expression of mutations and/or translocations supports the association of morphology with predictive biomarkers impacting on the therapeutical management.

The glucose metabolism was also essential for RAS-driven cancers as demonstrated in inducible mouse model for oncogenic *KRAS*G12D pancreatic duct adenocarcinoma (PDACs) [113]. In her review article, White described the results obtained by Ying demonstrating that RAS promotes glucose uptake and glycolysis even though tissue specific findings remain to be addressed and studied [42]. This determination represents a question mark especially because it is important to prove if this metabolic pathway may be attributed uniquely to PDACs or to other RAS-driven cancers [113]. It has been said that RAS transformation induces autophagy, which is required for tumor growth and mitochondrial function, whilst knockdown of autophagy genes causes impaired tumor progression [42].

Although *RET* proto-oncogene mutations are the most frequent mutation in medullary thyroid carcinoma (MTC), the presence of *RAS* mutations in sporadic MTC has been documented by the induction of MTCs in rascal transgenic mice with v-Ha-ras under the control of a specific promoter [116]. It has been reported that 0–41.2% and 0–40.9% of the cases had *HRAS* and *KRAS* mutations respectively, whilst and between 0–1.8% with *NRAS*. However to the best of our knowledge, the different type of *RET* and *RAS* mutation did not match with specific morphological features [117].

## CONCLUSIONS

The insights into the involvement of metabolic mechanisms of mutated-driven cancers seem to contribute to the morphological detection of several somatic mutations. Targeting these findings may shed light on new metabolic management and inhibitors as well as metabolic flaws. It makes sense that the well-documented role of Warburg effect is a relevant characteristic of several tumors, which may justify the morphological effects of these mechanisms. Highlighting these morphological findings will represent a significant additional aid to prove a possible involvement of the metabolic processes. The knowledge of these metabolic finding of *BRAF-*driven cancers may raise additional researches and questions postulating that somatic mutations induce organ-specific molecular mechanisms enabling different prognostic prediction in different cancers.

## References

[1] Rossi ED, Bizzarro T, Martini M, Capodimonti S, Fadda G, Larocca LM, Schmitt F (2014). The morphologic parameters able to predict BRAFV600E mutated malignancies on thyroid FNAC. Cancer Cytopathol.

[2] Rossi ED, Bizzarro T, Fadda G, Larocca LM, Schmitt F (2014). Is morphology alone able to predict BRAF mutated malignancies on thyroid FNAC?. Virchows Arch.

[3] Virk Virk RK, Theoharis CGA, Prasad A, Chhieng D, Prasad ML (2014). Morphology predicts BRAF V600E mutation in papillary thyroid carcinoma: an interobserver reproducibility study. Virchow Arch.

[4] Finkelstein A, Levy GH, Hui P, Prasad A Virk R, Chhieng DC, Carling T, Roman SA, Sosa JA, Udelsman R, Theoharis CG, Prasad ML Papillary thyroid carcinomas with and without BRAF V600E mutations are morphological distinct. Histopathol 2012; 60: 1052-59.

[5] Zeppernick F, Ardighieri L, Hannibal CG, Vang R, Junge J, Kjaer SK, Zhang R, Kurman RJ, Shih IeM BRAF mutation is associated with a specific cell type with features suggestive of senescence in ovarian serous borderline (atypical proliferative) tumors. Am J Surg Pathol 2014; 38: 1603-11.

[6] Ardighieri L, Zeppernick F, Hannibal CG, Vang R, Cope L, Junge J, Kjaer SK, Kurman RJ, Shih IeM (2014). Mutational analysis of BRAF and KRAS in ovarian atypical proliferative serous (borderline)tumors and associated peritoneal implants. J Pathol.

[7] Xing M (2007). Braf mutationin papillary thyroid cancer: pathogenic role, molecular bases and clinical implications. Endocr Rev.

[8] Oler G, Camacho CP, Hojaij FC, Michaluart P, Riggins GJ, Cerutti JM (2008). Gene expression profiling of papillary thyroid carcinoma identifies transcripts correlated with BRAF mutational status and lymph node metastasis. Clin Cancer Res.

[9] Rivera M, Ricarte-Filho J, Tuttle RM Ganly I, Shaha A, Knauf J, Fagin J, Ghossein R (2010). Molecular, morphologic and outcome analysis of thyroid carcinomas according to degree of extrathyroid extension. Thyroid.

[10] Kim SK, Kim DL, Han HS, Kim WS, Kim SJ, Moon WJ, Oh SY, Hwang TS (2008). Pyrosequencing analysis for detection of a BRAFV600E mutation in a FNAB specimen of thyroid nodules. Diagn Molecul Pathol.

[11] Yeo MK, Liang ZL, Oh T, An S, Kim MK, Kim KS, Shong M, Kim JM, Jo YS (2011). Pyrosequencing cut-off value identifying BRAF V600E mutation in fine needle aspiration samples of thyroid nodules. Clin Endocrinol.

[12] Hall A, Meyle KD, Lange MK, Klima M, Sanderhoff M, Dahl C, Abildgaard C, Thorup K, Moghimi SM, Jensen PB, Bartek J, Guldberg P, Christensen C (2013). Dysfunctional oxidative phosphorylation makes malignant melanoma cells addicted to glycolysis driven by the V600EBRAF oncogene. Oncotarget.

[13] Lee MH, Lee SE, Kim DW Ryu MJ, Kim SJ, Kim SJ, Kim YK, Park JH, Kweon GR, Kim JM, Lee JU, De Falco V, Jo YS, Shong M (2011). Mitochondrial localization and regulation of BRAF V600E in thyroid Cancer: a clinically used RAF inhibitor is unable to block the mitochondrial activities of BRAF V600E. J Clin Endocrinol Metab.

[14] Arozarena I, Sanchez-Laorden B, Packer L, Hidalgo-Carcedo C, Hayward R, Viros A, Sahai E, Marais R Oncogenic BRAF induces melanoma cell invasion by downregulating the cGMP-specific phosphodiesterase PDE5A. Cancer Cell.

[15] Johnson WT, Patel P, Hernandez A, Grandinetti LM, Huen AC, Marks S, Ho J, Monaco SE, Jaffe R, Picarsic J (2015). Langerhans cell histiocytosis and Erdheim-Chester disease, both with cutaneous presentations, and papillary thyroid carcinoma all harbouring the BRAFV600E mutation. J Cutan Pathol.

[16] Tiacci E, Pucciarini A, Bigerna B, Pettirossi V, Strozzini F, Martelli MP, Tabarrini A, Drexler HG, Falini B (2012). Absence of BRAFV600E in the human cell lines BONNA-12, ESKOL, HAIR-M and HC-1 questions their origin from hairy cell leukemia. Blood.

[17] Balsat M, Cornillon J (2013). Molecular and therapeutic advances in hairy cell leukemia. Bull Cancer.

[18] Landau MS, Kuan SF, Chiosea S, Pai RK Braf-mutated microsatellite stable colorectal carcinoma: An aggressive adenocarcinoma with reduced CDX2 and increased cytokeratin 7 immunohistochemical expression. Hum Pathol.

[19] Tajima S, Ohata A, Koda K, Maruyama Y (2015). Myxoid epithelioid gastrointestinal stromal tumor harboring an unreported PDGFRA mutation: report of a case and review of the literature. Int J Clin Pathol.

[20] Warth A, Penzel R, Lindenmaier H, Brandt R, Stenzinger A, Herpel E, Goeppert B, Thomas M, Herth FJ, Dienemann H, Schnabel PA, Schirmacher P, Hoffmann H EGFR, KRAS, BRAF and ALK gene alterations in lung adenocarcinomas: patient outcome, interplay with morphology and immunophenotype. Eur Resp J.

[21] Trovisco V, Vieira de Castro I, Soares P, Máximo V, Silva P, Magalhães J, Abrosimov A, Guiu XM, Sobrinho-Simões M BRAF mutations are associated with some histological types of papillary thyroid carcinoma. J Pathol.

[22] Jung CK, Little MP, Lubin JH, Brenner AV, Wells SA, Sigurdson AJ, Nikiforov YE (2014). The increase in thyroid cancer incidence during the last four decades is accompanied by a high frequency of BRAF mutations and a sharp increase in RAS mutations. J Clin Endocrinol Metab.

[23] Mathur A, Moses W, Rahbari R, Khanafshar E, Duh QY, Clark O, Kebebew E (2011). Higher rate of BRAF mutation in papillary thyroid cancer over time: A single institution study. Cancer.

[24] Kim TH, Park YJ, Lim JA, Ahn HY, Lee EK, Lee YJ, Kim KW, Hahn SK, Youn YK, Kim KH, Cho BY, Park do J The association of the BRAFV600E mutation with prognostic factors and poor clinical outcome in papillary thyroid cancer. Cancer.

[25] Basolo F, Torregrossa L, Giannini R, Miccoli M, Lupi C, Sensi E, Berti P, Elisei R, Vitti P, Baggiani A, Miccoli P Correlation between the BRAF V600E mutation and tumor invasiveness in papillary thyroid carcinomas smaller tha 20 millimeters: analysis of 1060 cases. J Clin Endocrinol Metab.

[26] Lupi C, Giannini R, Ugolini C, Proietti A, Berti P, Minuto M, Materazzi G, Elisei R, Santoro M, Miccoli P, Basolo F Association of BRAF V600E mutation with poor clinical-pathological outcoms in 500 consecutive cases of papillary thyroid carcinoma. J Clin Endocrinol Metab.

[27] Guerra A, Fugazzola L, Marotta V, Cirillo M, Rossi S, Cirello V, Forno I, Moccia T, Budillon A, Vitale M A high percentage of BRAFV600E alleles in papillary thyroid carcinoma predicts a poorer out come. J Clin Endocrinol Metab.

[28] Schulten HJ, Salama S, Al-Mansouri Z, Alotibi R, Al-Ghamdi K, Al-Hamour OA, Sayadi H, Al-Aradati H, Al-Johari A, Huwait E, Gari M, Al-Qahtani MH, Al-Maghrabi J BRAF mutations in thyroid tumors from an ethnically diverse group. Hered Cancer Clin Pract.

[29] Jung CK, Im SY, Kang YJ, Lee H, Jung ES, Kang CS, Bae JS, Choi YJ Mutational patterns and novel mutations of the BRAF gene in a large cohort of Korean patients with papillary thyroid carcinoma. Thyroid.

[30] Barollo S, Pezzani R, Cristiani A, Redaelli M, Zambonin L, Rubin B, Bertazza L, Zane M, Mucignat-Caretta C, Bulfone A, Pennelli G, Casal Ide E, Pelizzo MR Prevalence, tumorigenic role, and biochemical implications of rare BRAF alterations. Thyroid.

[31] Nikiforova MN, Nikiforov Y (2009). Molecular diagnostics and predictors in thyroid cancer. Thyroid.

[32] Nikiforov YE Molecular diagnostics of thyroid tumors. Archives of Pathology and Laboratory medicine.

[33] Yip L, Nikiforova MN, Carty SE, Yim JH, Stang MT, Tublin MJ, Lebeau SO, Hodak SP, Ogilvie JB, Nikiforov YE Optimizing surgical treatment of papillary thyroid carcinoma associated with BRAF mutation. Surgery 2009; 146:1215-23.

[34] Nikiforov YE, Steward DL, Robinson-Smith TM, Haugen BR, Klopper JP, Zhu Z, Fagin JA, Falciglia M, Weber K, Nikiforova MN Molecular testing for mutations in improving the fine needle aspiration diagnosis of thyroid nodules. J Clin Endocr Metab.

[35] Ohori NP, Nikiforova MN, Schoedel KE, LeBeau SO, Hodak SP, Seethala RR, Carty SE, Ogilvie JB, Yip L, Nikiforov YE Contribution of molecular testing to thyroid fine needle aspiration cytology of “Follicular lesion of undetermined significance/Atypia of undetermined significance”. Cancer Cytopathol.

[36] Elisei R, Ugolini C, Viola D, Lupi C, Biagini A, Giannini R, Romei C, Miccoli P, Pinchera A, Basolo F (2008). BRAF mutation and outcome of patients with papillary thyroid carcinoma: a 15-year median follow-up study. J.Clin.Endocrinol.Metab.

[37] Rossi ED, Martini M, Capodimonti S, Straccia P, Cenci T, Lombardi CP, Pontecorvi A, Larocca LM, Fadda G (2013). Diagnostic and prognostic value of immunocytochemistry and BRAF mutation analysis on liquid based biopsies of thyroid neoplasms suspicious for carcinoma. Eur J Endocrin.

[38] Rossi ED, Martini M, Capodimonti S, Lombardi CP, Pontecorvi A, Vellone VG, Zannoni GF, Larocca LM, Fadda G (2013). BRAF (V600E) mutation analysis on LBC-processed aspiration biopsies predicts bilaterality and nodal involvement in papillary thyroid microcarcinoma. Cancer Cytopathol.

[39] Gandolfi G, Sancisi V, Piana S, Ciarrocchi A (2015). Time to re-consider the meaning of BRAF V600E mutation in papillary thyroid carcinoma. Int J Cancer.

[40] McCarthy N (2015). Mutant BRAF feels the burn. Nature Review.

[41] Kang HB, Fan J, Lin R, Elf S, Ji Q, Zhao L, Jin L, Seo JH, Shan C, Arbiser JL, Cohen C, Brat D, Miziorko HM Metabolic rewiring by oncogenic BRAFV600E links ketogenesis pathway to BRAF-MEK1. Mol Cell.

[42] White E (2015). Exploiting the bad eating habits of RAS-driven cancers. Genes and Develop.

[43] Strohecker AM, White E (2014). Targeting mitochondrial metabolism by inhibiting autophagy in BRAF-driven cancers. Cancer Discov.

[44] Pinheiro C, Penna V, Morais-Santos F, Abrahao-Machado LF, Ribeiro G, Curcelli EC, Olivieri MV, Morini S, Velnca I, Ribeiro D, Schmitt F, Reis RM, Baltazar F (2014). Characterization of monocarboxylate transporters (MCTs) expression in soft tissue sarcomas: distinct prognostic impact of MCT1 subcellular localization. J Transl Med.

[45] Pinheiro C, Longatto-Filho A, Scapulatempo C, Ferreira L, Martins S, Pellerin L, Rodrigues M, Alves VAF, Schmitt F, Baltazar F Increased expression of monocarboxylate transporters 1,2 and 4 in colorectal carcinomas. Virchows Arch.

[46] Pinheiro C, Longatto-Filho A, Simoes K, Jacob CE, Bresciani CJC, Zilberstein B, Cecconello I, Alves VAF, Schmitt F, Baltazar F The prognostic value of CD147/EMMPRIN is associated with monocarboxylate transporter 1 co-expression in gastric cancer. Eur J Cancer.

[47] Pinheiro C, Albergaria A, Paredes J, Sousa B, Dufloth R, Vieira D, Schmitt F, Baltazar F (2010). Monocarboxylate transporter 1 is up-regulated in basal-like breast carcinoma. Histopathol.

[48] Pinheiro C, Longatto-Filho A, Soares T, Pereira H, Bedrossian C, Michael C, Schmitt FC, Baltazar F CD147 immunohistochemistry discriminates between reactive mesothelial cells and malignant mesothelioma. Diagn Cytopathol.

[49] Pinheiro C, Longatto-Filho A, Pereira SMM, Etlinger D, Moreira MAR, Jube’ LF, Queiroz GS, Schmitt FC, Baltazar F Monocarboxylate transporters 1 and 4 are associated with CD147 in cervical carcinoma. Disease MARKERS.

[50] Narumi K, Furugen A, Kobayashi M, Otake S, Itagaki S, Iseki K Regulation of monocarboxylate transporter 1 in skeletal muscle cells by intracellular signaling pathways. Biol Pharm Bull.

[51] Davies H, Bignell GR, Cox C (2002). Mutations of the BRAF gene in human cancer. Nature.

[52] Rossi ED, Bizzaro T, Granja S, Martini M, Capodimonti S, Luca E, Fadda G, Lombardi CP, Pontecorvi A, Larocca LM, Baltazar F, Schmitt F (2016). The expression of monocarboxylate transporters in thyroid carcinoma can be associated with the morphological features of BRAFV600E mutation. Endocrine.

[53] Merritt MA, Cramer DW (2010). Molecular pathogenesis of endometrial and ovarian cancer. Cancer Biomark.

[54] Mayr D, Hirschmann A, Löhrs U, Diebold J (2006). KRAS and BRAF mutations in ovarian tumors: a comprehensive study of invasive carcinomas, borderline tumors and extraovarian implants. Gynecol Oncol.

[55] Combe P, Chauvenet L, Lefrère-Belda MA, Blons H, Rousseau C, Oudard S, Pujade-Lauraine E (2015). Sustained response to vemurafenib in a low grade serous ovarian cancer with a BRAF V600E mutation. Invest New Drugs.

[56] Della Pepa C, Tonini G2, Santini D2, Losito S3, Pisano C4, Di Napoli M4, Cecere SC4, Gargiulo P5, Pignata S4 (2015). Low Grade Serous Ovarian Carcinoma: from the molecular characterization to the best therapeutic strategy. Cancer Treat Rev.

[57] Preusser M, Capper D, Berghoff AS, Horvat R, Wrba F, Schindl M, Schoppmann SF, von Deimling A, Birner P (2013). Expression of BRAF V600E mutant protein in epithelial ovarian tumors. Appl Immunohistochem Mol Morphol.

[58] Bösmüller H, Fischer A, Pham DL, Fehm T, Capper D, von Deimling A, Bonzheim I, Staebler A, Fend F (2013). Detection of the BRAF V600E mutation in serous ovarian tumors: a comparative analysis of immunohistochemistry with a mutation-specific monoclonal antibody and allele-specific PCR. Hum Pathol.

[59] Vang R, Shih IeM, Kurman RJ (2009). Ovarian low-grade and high-grade serous carcinoma: pathogenesis, clinicopathologic and molecular biologic features, and diagnostic problems. Adv Anat Pathol.

[60] Kurman RJ (2013). Origin and molecular pathogenesis of ovarian high-grade serous carcinoma. Ann Oncol.

[61] Vereczkey I, Serester O, Dobos J, Gallai M, Szakács O, Szentirmay Z, Tóth E (2011). Molecular characterization of 103 ovarian serous and mucinous tumors. Pathol Oncol Res.

[62] Salama AKS, Flaherty KT (2013). Braf in melanoma: current strategies and future directions. Clin Cancer Res.

[63] Kim SY, Kim SN, Hahn HJ (2015). Metaanalysis of BRAF mutations and clinicopathologic characteristics in primary melanoma. J Am Ac Derm.

[64] Goodall J, Wellbrock C, Dexter TJ (2004). The brn2 transcription factor links activated BRAF to melanoma proliferation. Mol Cell Biol.

[65] Pinner S, Jordan P, Sharrock K (2009). Intravital imaging reveals transiet changes in pigment production and BRN2 expression during metastatic melanoma dissemination. Cancer.

[66] Haq R, Fisher DE, Widlund HR (2014). Molecular pathways: BRAF induces bioenergetic adaptation by attenuating oxidative phosphorylation. Clin Cancer Res.

[67] Haq R, Shoag J, Andreu-Perez P, Yokoyama S, Edelman H, Rowe GC, Frederick DT, Hurley AD, Nellore A, Kung AL, Wargo JA, Song JS, Fisher DE (2013). Oncogenic BRAF regulates oxidative metabolism via PGC1α and MITF. Cancer Cell.

[68] Wellbrock C, Rana S, Paterson H, Pickersgill H, Brummelkamp T, Marais R (2008). Oncogenic BRAF regulates melanoma proliferation through the lineage specific factor MITF. PLoS One.

[69] Hartman ML, Czyz M (2015). MITF in melanoma: mechanisms behind its expression activity. Cell Mol Life Sci.

[70] Wellbrock C, Marais R (2005). Elevated expression of MITF counteracts B-RAF-stimulated melanocyte and melanoma cell proliferation. Cell Biol.

[71] Abildgaard C (2015). Molecular drivers of cellular metabolic reprogramming in melanoma. Trends Mol Med.

[72] Murata T, Shimizu K, Watanabe Y, Morita H, Sekida M, Tagawa T (2010). Expression and role of phosphodiesterase 5 in human malignant melanoma cell line. Anticancer Res.

[73] Viale A, Cori D, Draetta GF (2015). Tumors and mitochonrial repsiration: A neglected connection. Cancer Res.

[74] Lakhter AJ, Hamilton J, Dagher PC, Mukkamala S, Hato T, Dong XC, Mayo LD, Harris RA, Shekhar A, Ivan M, Brustovetsky N, Naidu SR (2014). Ferroxitosis: a cell death from modulation of oxidative phosphorylation and PKM2-dependent glycolysis in melanoma. Oncotarget.

[75] Davies H, Bignell GR, Cox C, Stephens P, Edkins S, Clegg S (2002). Mutations of the BRAF gene in human cancer. Nature.

[76] Pakneshan S, Salajegheh A, Smith RA, Lam AKY (2013). Clinicopathological relevance of BRAF Mutation in human cancer. Pathol.

[77] Machnicki MM, Stokloza T (2014). BRAF-a new player in haematological neoplasms. Blood Cell, Mol Dis.

[78] Gustafsson B, Angelini S, Sander B, Christensson B, Hemminki K, Kumar R (2005). Mutations in the BRAF and N-ras genes in childhood acute lymphoblastic leukemia. Leukemia.

[79] Andrulis M, Lehners N, Capper D, Penzel R, Heining C, Huellein J, Zenz T, von Deimling A, Schirmacher P, Ho AD, Goldschmidt H, Neben K, Raab MS (2013). Targeting the BRAF V600E mutation in multiple myeloma. Cancer Discov.

[80] Domingo E, Schwartz SJ RAF (v-raf murine sarcoma viral oncogene homolog B1) Atlas Gent Cytogenet Oncol Haematol. 2004.

[81] Langabeer SE, Quinn F, O’Brien McElligott AM, Kelly J, Browne PV, Vandenberghe E (2012). Incidence of the BRAFV600E mutation in chronic lymphocytic leukemia and prolymphocytic leukemia. Leuk Res.

[82] Jebarai BM, Kienle D, Buhler A, Winkler D, Döhner H, Stilgenbauer S, Zenz T (2013). RAF mutations in chronic lymphocytic leukemia. Leuk Lymphoma.

[83] Lee JW, Soung YH, Park WS, Kim SY, Nam SW, Min WS, Lee JY, Yoo NJ, Lee SH (2004). BRAF mutations in acute leukemias. Leukemia.

[84] Bonello L, Voena C, Ladetto M, Boccadoro M, Palestro G, Inghirami G, Chiarle R (2003). BRAF gene is not mutated in plasma cell leukemia and multiple myeloma. Leukemia.

[85] Chapman MA, Lawrence MS, Keats JJ, Cibulskis K, Sougnez C, Schinzel AC, Harview CL, Brunet JP, Ahmann GJ, Adli M, Anderson KC, Ardlie KG, Auclair D (2011). Initial genome sequencing and analysis of multiple mieloma. Nature.

[86] Blombery PA, Wong SQ, Hewitt CA, Dobrovic A, Maxwell EL, Juneja S, Grigoriadis G, Westerman DA (2012). Detection of BRAF mutations in patients with hairy cell leukemia and related lymphoproliferative disorders. Haematologica.

[87] Jones G, Parry-Jones N, Wilkins B, Else M, Catovsky D (2012). British Committee for Standards in Haematology Revised guidelines for the diagnosis and management of hairy cell leukemia and hairy cell leukemia variant. Br J Haematol.

[88] Ahmadzadeh A, Shahrabi S, Jaseb K, Norozi F, Shahjahani M, Vosoughi T, Hajizamani S, Saki N (2014). BRAF mutation in hairy cell leukemia. Oncol Rev.

[89] Tiacci E, Park JH, De Carolis L, Chung SS, Broccoli A, Scott S, Zaja F, Devlin S, Pulsoni A, Chung YR, Cimminiello M, Kim E, Rossi D (2015). Targeting mutant RAF in relapsed or refractory hairy-cell leukemia. N Engl J Med.

[90] Langabeer SE, Brien DO, Liptrot S, Flynn CM, Hayden PJ, Conneally E, Browne PV, Vandenberghe E (2012). Correlation of the BRAF V600E mutation in hairy cell leukemia with morphology, cytochemistry and immunophenotype. Int J. Lab Hem.

[91] Quest GR, Johnston JB (2015). Clinical features and diagnosis of hairy cell leukemia. Pract Res Clin Hematol.

[92] Abla O, Egeler RM, Weitzman S (2010). Langerhans cell histiocytosis: current concepts and treatments. Cancer Treat Rev.

[93] Badalian-Very G, Vergilio JA, Degar BA, Rodriguez-Galindo C, Rollins BJ (2012). Recent advances in the understanding of Langerhans cell histiocytosis. Br J Haematol.

[94] Ng-Cheng-Hin B, O’Hanlon-Brown C, Alifrangis C, Waxman J (2011). Langerhans cell histicytosis: Old disease new treatment. QJM.

[95] Willman CL, Busque L, Griffith BB, Favara BE, McClain KL, Duncan MH, Gilliland DG (1994). Langerhans's-cell histiocytosis(histiocytosis X)-a clonal proliferative disease. N Engl J Med.

[96] Sahm F, Capper D, Preusser M, Meyer J, Stenzinger A, Lasitschka F, Berghoff AS, Habel A, Schneider M, Kulozik A, Anagnostopoulos I, Müllauer L, Mechtersheimer G (2012). BRAF V600E mutat protein is expressed in cells of variable maturation in Langerhans cells histiocytosis. Blood.

[97] Mazor RD, Kesier A, Shoenfeld Y (2012). Erdheim-Chester disease: an orphan condition seeking treatment. Isr. Med Assoc J.

[98] Veysser-Belot C, Cacoub P, Caparros-Lefebvre D, Wechsler J, Brun B, Remy M, Wallaert B, Petit H, Grimaldi A, Wechsler B, Godeau P (1996). Erdheim-Chester disease. Clinical and radiologic characteristics of 59 cases. Medicine (Baltimore).

[99] Chetritt J, Paradis V, Dargere D, Adle-Biassette H, Maurage CA, Mussini JM, Vital A, Wechsler J, Bedossa P (1999). Erdheim-Chester disease: a neoplastic disorder. Hum Pathol.

[100] Haroche J, Cohen-Aubart F, Emile JF, Arnaud L, Maksud P, Charlotte F, Cluzel P, Drier A, Hervier B, Benameur N, Besnard S, Donadieu J, Amoura Z (2013). Dramatic efficacy of vemurafenib in both multisystemic and refractory Erdheim-Chester disease and Langerhans cell histiocytosis harboring the BRAFV600E mutation. Blood.

[101] Dougherty MJ, Santi M, Brose MS, Ma C, Resnick AC, Sievert AJ, Storm PB, Biegel JA (2010). Activating mutations in BRAF characterize a spectrum of pediatric low-grade gliomas. Neuro Oncol.

[102] Schiffman JD, Hodgson JG, VandenBerg SR, Flaherty P, Polley MY, Yu M, Fisher PG, Rowitch DH, Ford JM, Berger MS, Ji H, Gutmann DH, James CD (2010). Oncogenic BRAF mutation with CDKN2A inactivation is characteristic of a subset of pediatric malignant astrocytomas. Cancer Res.

[103] Dias-Santagata D, Lam Q, Vernovsky K, Vena N, Lennerz JK, Borger DR, Batchelor TT, Ligon KL, Iafrate AJ, Ligon AH, Louis DN, Santagata S (2011). BRAF V600E Mutations Are Common in Pleomorphic Xanthoastrocytoma: Diagnostic and Therapeutic Implications. PLoS One.

[104] Lee D, Cho YH, Kang SY, Yoon N, Sung CO, Suh YL (2015). BRAF V600E mutations are frequent in dysembryoplastic neuroepithelial tumors and subependymal giant cell astrocytomas. J Surg Oncol.

[105] MacConaill LE, Campbell CD, Kehoe SM, Bass AJ, Hatton C, Niu L, Davis M, Yao K, Hanna M, Mondal C, Luongo L, Emery CM, Baker AC (2009). Profiling critical cancer gene mutations in clinical tumor samples. PLoS ONE.

[106] Carr NJ, Mahajan H, Tan KL, Hawkins NJ, Ward RL (2009). Serrated and non-serrated polyps of the colorectum: their prevalence in an unselected case series and correlation of BRAF mutation analysis with the diagnosis of sessile serrated adenoma. J Clin Pathol.

[107] Kim KM, Lee EJ, Ha S, Kang SY, Jang KT, Park CK, Kim JY, Kim YH, Chang DK, Odze RD (2011). Molecular features of colorectal hyperplastic polyps and sessile serrated adenoma/polyps from KoreaAm. J Surg Pathol.

[108] Yang HM1, Mitchell JM, Sepulveda JL, Sepulveda AR (2015). Molecular and histologic considerations in the assessment of serrated polyps. Arch Pathol Lab Med.

[109] Rosty C1, Buchanan DD, Walsh MD, Pearson SA, Pavluk E, Walters RJ, Clendenning M, Spring KJ, Jenkins MA, Win AK, Hopper JL, Sweet K, Frankel WL (2012). Phenotype and polyp landscape in serrated polyposis syndrome: a series of 100 patients from genetics clinics. Am J Surg Pathol.

[110] Mesteri I, Bayer G, Meyer J, Capper D, Schoppmann SF, von Deimling A, Birner P (2014). Improved molecular classification of serrated lesions of the colon by immunohistochemical detection of BRAF V600E. Mod Pathol.

[111] Rosembaum MW, Bledsoe JR, Morales-Oyarvide V, Huynh TG, Mino-Kenudson M (2016). PD-L1 expression in colorectal cancer is associated with microsatellite instability, BRAF mutation, Medullary morphology and cytotoxic tumor-infiltrating lymphocytes. Mod Pathol.

[112] Kim HR, Shim HS, Chung JH, Lee YJ, Hong YK, Rha SY, Kim SH, Ha SJ, Kim SK, Chung KY, Soo R, Kim JH, Cho BC (2012). Distinct clinical features and utcomes in never-smokes with nonsmall cell lung cancer who harbor EGFR or KRAS mutations or ALK rearrangement. Cancer.

[113] Ying H, Kimmelman AC, Lyssiotis CA, Hua S, Chu GC, Fletcher-Sananikone E, Locasale JW, Son J, Zhang H, Coloff JL, Yan H, Wang W, Chen S (2012). Oncogenic KRAS maintains pancreatic tumors through regulation of anabolic glucose metabolism. Cell.

[114] Debiec-Rychter M, Wasag B, Stul M, De Wever I, Van Oosterom A, Hagmeijer A, Sciot R (2004). Gastrointestinal stromal tumors (GISTs) negative for KIT (CD117 antigen) immunoreactivity. J Pathol.

[115] Hirota S, Ohashi A, Nishida T, Isozaki K, Kinoshita K, Shinomura Y, Kitamura Y (2003). Gain-of-function mutations of platelet-derived growth factor receptor alpha gene in gastrointestinal stromal tumors. Gastroenterology.

[116] Moura MM, Cavaco BM, Leite V (2015). RAS-protooncogene in medullary thyroid carcinoma. Endocr Relat Cancer.

[117] Tamburrino A, Molinolo AA, Salerno P, Chernock RD, Raffeld M, Xi L, Gutkind JS, Moley JF, Wells SA, Santoro M (2012). 2012 Activation of the mTOR pathway in primary medullary thyroid carcinoma and lymph node metastases. Clinical Cancer Research.

